# Changes in the Volatile Profile, Fruity Flavor, and Rancidity in Virgin Olive Oils During Storage by Targeted and Untargeted Analytical Approaches

**DOI:** 10.3390/foods14111884

**Published:** 2025-05-26

**Authors:** Rosalba Tucci, Chiara Cevoli, Alessandra Bendini, Sara Barbieri, Enrico Casadei, Enrico Valli, Tullia Gallina Toschi

**Affiliations:** 1 Department of Agricultural and Food Science, Alma Mater Studiorum-Università di Bologna, 47521 Cesena, Italy; rosalba.tucci2@unibo.it (R.T.); chiara.cevoli3@unibo.it (C.C.); sara.barbieri@unibo.it (S.B.); enrico.casadei15@unibo.it (E.C.); enrico.valli4@unibo.it (E.V.); tullia.gallinatoschi@unibo.it (T.G.T.); 2 Interdepartmental Center for Industrial Agrofood Research, Alma Mater Studiorum-Università di Bologna, 47521 Cesena, Italy

**Keywords:** virgin olive oil, storage, quality grade, HS-GC–IMS, SPME-GC–MS, FGC, volatile compounds, chemometric analysis, sensory analysis

## Abstract

The changes in monovarietal extra virgin olive oils (EVOOs), produced with olives grown under different agronomic conditions, were investigated by targeted and untargeted analytical approaches. Specifically, volatile molecules were monitored in oils just produced and stored for 6 and 12 months with two different packaging solutions. The targeted SPME-GC–MS method showed an increase in volatile markers of lipid oxidation. Moreover, more rapid analytical approaches, namely targeted HS-GC–IMS and untargeted FGC, were used to investigate volatile organic compounds (VOCs). These chromatographic methods, respectively, returned heatmaps and fingerprint profiles that were elaborated on by multivariate analysis. Exploratory principal component analysis performed on the data from VOCs allowed the clustering of samples based on the storage time. The quality of samples was also determined by a panel test. Furthermore, this study employed previously built models using partial least squares discriminant analysis to confirm the sensory classification of the stored samples. Based on these predictive models, all samples were confirmed as EVOO, except for one categorized as virgin (rancid according to the panel test). This classification was further supported by the SPME-GC–MS analysis, which revealed higher concentrations of lipid oxidation markers in this specific sample, in particular the (*E*)-2-heptenal reached a concentration twenty times higher than its odor threshold. In addition, five oils were inconsistently classified by the models and considered at risk of downgrading the commercial category after 12 months of storage.

## 1. Introduction

European food safety regulations require almost all food products to display a date indicating their suitability for consumption [1]. For perishable foods such as milk and meat, the label must include an expiration date. In contrast, products with a longer shelf life and low safety risk during storage, such as virgin olive oils, are labeled with a “best before” date. This minimum durability date indicates the timeframe during which the extra virgin olive oil (EVOO) retains its chemical, physical, and organoleptic characteristics when stored appropriately. Failure to meet these standards constitutes food fraud due to regulatory non-compliance [2]. Producers are responsible for determining this durability period, which typically ranges between 12 and 18 months after bottling [3,4].

Beyond regulatory compliance, this labeling serves an important commercial function by offering consumers, food industry stakeholders, and regulatory bodies a guarantee of the product’s quality. This practice enhances brand trust and strengthens customer loyalty [5]. According to Reg. EC 2022/2104, it is possible to distinguish three commercial categories of virgin olive oils: extra virgin, virgin (VOO), and lampante (LOO) [3]. The latter is not suitable for consumption in its current form and must be refined to make it appropriate for edible use [6].

EVOO is considered among the highest quality vegetable oils, which also implies a superior sensory quality, and is appreciated for its nutritional value due to its favorable content in monounsaturated fatty acids and antioxidants [7]. Consequently, EVOO is characterized by a high price on the market compared with other vegetable oils [8]. Many quality indicators of EVOO outlined in food legislation can change during the product’s shelf life, in particular due to oxidative degradation.

Several factors influence the lipid oxidation of EVOO, including the quality of the olives before milling, the extraction techniques used, and the level of exposure to pro-oxidative agents, mainly oxygen, light, and temperature [9]. In particular, the concentration of antioxidants like tocopherol and secoiridoid derivatives can be reduced over time, significantly affecting the shelf life of samples [10,11]. Furthermore, the decomposition of hydroperoxides of fatty acids, primary oxidation products, leads to the formation of new volatile organic compounds (VOCs) as secondary oxidation products that are responsible for the development of off-flavors and a decrease in product quality [12].

Therefore, the identification and quantification of VOCs directly related to positive (such as fruity notes) and negative attributes (like rancid defect) are of great importance to assess the quality of VOOs [13]. Sensory analysis, through the detection of the rancid defect, represents a valid method to monitor the quality of VOOs during storage [11]. In the literature, various analytical solutions have been developed to support sensory analysis and evaluate the volatile profile of VOOs. Traditionally, solid-phase microextraction (SPME) coupled with gas chromatography–mass spectrometry (GC–MS) or flame ionization detection (FID) has been extensively applied to the identification and quantification of volatile compounds in olive oils [14,15,16,17,18].

In recent years, static headspace analysis associated with techniques such as gas chromatography–ion mobility spectrometry (GC–IMS) and flash gas chromatography (FGC) has provided new opportunities for the rapid screening of olive oils [19,20,21,22].

This study aims to analyze the evolution of headspace volatile profiles and sensory attributes in monovarietal VOOs produced from “Nostrana di Brisighella” cv. olives that were grown under different agronomical conditions and picked at maturity indices, over a 12-month storage period. An integrated analytical approach, combining a panel test, SPME-GC–MS, HS-GC–IMS, and FGC techniques, was employed to investigate changes in the volatile profiles, with a particular focus on VOCs associated with oxidative processes and sensory changes.

## 2. Materials and Methods

### 2.1. Reagents

The standards used to analyze VOCs were purchased from Sigma-Aldrich (St. Louis, MO, USA) and are listed below, along with their corresponding CAS numbers and purity: ethanol (64-17-5, ≥99.9%), hexanal (66-25-1, ≥98.0%), (*E*)-2-hexenal (6728-26-3, ≥97.0%), (*Z*)-3-hexenyl acetate (3681-71-8, ≥98.0%), 1-hexanol (111-27-3, ≥99.9%), nonanal (124-19-6, ≥95.0%), 1-octen-3-ol (3391-86-4, ≥98.0%), acetic acid (64-19-7, ≥99.8%), octane (111-65-9, ≥99.7%), ethyl acetate (141-78-6, ≥99.8%), ethyl propanoate (105-37-3, ≥99.7%), 3-methyl-1-butanol (123-51-3, ≥98.5%), (*E*)-2-heptenal (18829-55-5, ≥95%), 6-methyl-5-hepten-2-one (110-93-0, ≥97.0%), (*E*,*E*)-2,4-hexadienal (142-83-6, ≥95.0%), propionic acid (79-09-4, ≥99.8%), (*E*)-2-decenal (3913-81-3, ≥95.0%), and pentanoic acid (109-52-4, ≥99.8%). 4-Methyl-2-pentanol (123-51-3, ≥95%) was used as internal standard (IS), while a mixture of *n*-alkanes (C8–C20, ~40 mg/L each, in *n*-hexane) was employed to calculate the linear retention indices (LRI).

### 2.2. Olive Oil Samples

**Figure 1:**
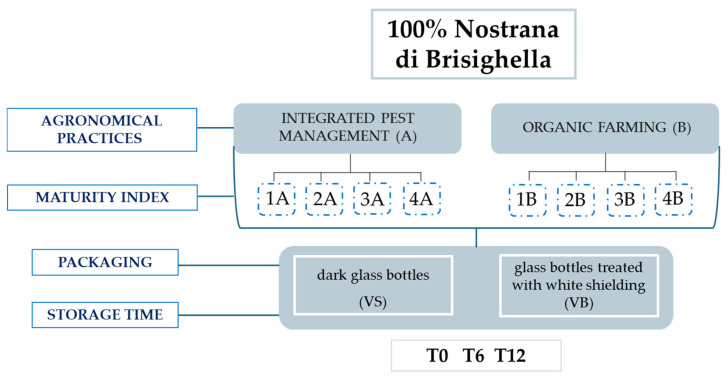
Agronomic systems and technological parameters considered in the sampling plan carried out during the 2022/23 olive oil campaign.

This study was carried out on 32 monovarietal VOOs produced from the olive variety “Nostrana di Brisighella”. The olives were harvested from olive groves in a circumscribed area of the Emilia-Romagna region (Italy) according to the production specifications for Brisighella PDO. Two different agronomic systems were employed: integrated pest management and organic farming. Olives were harvested during the 2022/2023 olive oil campaign, from 10 October to 15 November 2022. This period was divided into four weeks, during which olives were collected at four different maturity indices (MI) from both systems (see values in [23]): integrated pest management system, coded as A, and organic farming system, coded as B (Figure 1). The oil samples were produced on the same day as the harvest, ensuring uniform production conditions by an industrial mill. Exclusively for samples from olives harvested in the first and fourth weeks, bottling was conducted using two types of 0.75 L packaging: classic dark glass bottles (coded as VS) and glass bottles treated with white shielding (coded as VB). All other samples were stored in dark glass bottles. The samples were kept on shelves in a room, with exposure to natural light and a temperature close to 25 °C, for 12 months, with periodic analyses performed at the 6-month mark.

### 2.3. Sensory Analysis

The sensory evaluation of the samples was conducted by the Professional Committee of DISTAL (Department of Agricultural and Food Sciences, Alma Mater Studiorum-Università di Bologna), which has been recognized by the Italian Ministry of Agriculture, Food Sovereignty, and Forestry since 2006. The assessment focused on the main sensory defects and the three main positive attributes of VOOs (fruity, bitter, and pungent) using continuous 10 cm scales to determine the median intensity of each attribute, in accordance with official procedures [3,4]. Based on these medians, oils were classified as “robust” (median > 6.0), “medium” (median > 3.0 and ≤6.0), or “delicate” (median ≤ 3.0). Additional positive attributes were included in the profile sheet according to the list of descriptors required for the designation of origin (PDO) for EVOOs, as established by the International Olive Council (IOC) standards [24].

### 2.4. Sample Preparation for Headspace Analysis

The samples were analyzed in duplicate by HS-GC–IMS and FGC analytical techniques by introducing an aliquot of VOO into a hermetically closed 20 mL glass vial and working in a lab at room temperature. For the preparation of samples and IS mixture for SPME-GC–MS analysis the method described by Casadei et al., 2021 and Aparicio et al., 2022 was followed, with some modifications [25,26]. Specifically, 1.9 g of sample was weighed in a 20 mL glass vial and 0.1 g of 4-methyl-2-pentanol solution in refined olive oil (50 mg/kg) was added as IS. The vial was hermetically closed with a polytetrafluoroethylene septum. Each sample was analyzed in triplicate.

### 2.5. HS-GC–IMS Analysis of VOO Samples

As detailed by Valli et al., 2020, HS-GC–IMS analysis was performed using the HS-GC–IMS Flavourspec^®^ instrument (G.A.S. Dortmund, Dortmund, Germany) coupled with a nitrogen generator (Microprogel, Pordenone, Italy) [27]. For each sample, the headspace (100 µL) was injected in a splitless mode by the HT2000H autosampler (HTA S.R.L., Brescia, Italy) into a heated injector. The analytes were separated, initially, into a low-polar column FS-SE-54-CB-0.5 and then analyzed by IMS equipped with a tritium ionizing radioactive source (5000 V) and a 9.8 cm long drift tube (Gesellschaft für Analytische Sensorsysteme mbH, G.A.S.; Dortmund, Germany). The samples were analyzed according to the analytical parameters previously published by Valli et al., 2020 [27]. A 3D chromatogram heatmap was obtained for each sample, highlighting only the areas corresponding to the 15 volatile markers (monomers and/or dimers) of interest. This procedure, outlined by Valli et al., 2020 [27] with minor modifications, focuses on 15 volatile compounds that are considered the minimum number of relevant markers associated with fruity and sensory negative attributes in VOOs, considering the literature [28,29]. A total of 24 values of signal intensities (monomer and/or dimer) was exported using VOCal software version 0.4.07 (Gesellschaft für Analytische Sensorsysteme mbH, G.A.S.; Dortmund, Germany), and the final data matrix (32X24) was used for data elaboration.

### 2.6. SPME-GC–MS Analysis of VOO Samples

VOCs analysis was carried out using SPME-GC–MS following the preparation step and chromatographic conditions described in Aparicio et al., 2022, with a different quantification strategy [26]. Each vial was conditioned at 40 °C for 10 min under agitation. Successively, a 1 cm DVB/CAR/PDMS fiber (50/30 μm; Supelco Ltd., Bellefonte, PA, USA) was inserted into the vial and exposed to headspace for 40 min at 40 °C using an autosampler (AOC-5000 plus, Shimadzu, Kyoto, Japan). The VOCs adsorbed on the fiber were desorbed in the injector port for 5 min at 250 °C in split mode (1:10). GC–MS analysis was performed using the QP2010 Ultra system (Shimadzu, Kyoto, Japan) equipped with a TG-WAXMS capillary column (60 m, internal diameter 0.25 mm ID, and coating 0.50 μm; Thermo Fisher Scientific, Waltham, MA, USA). The GC–MS analytical conditions, including the oven temperature program and detector settings, followed those described by Casadei et al., 2024 [23]. The tentative identification of the volatile compounds by SPME-GC–MS was performed by MS comparing analyte spectra with reference spectra from the NIST library (2008 version). In addition, the LRI of each compound was calculated (C8 to C20 *n*-alkane series) and compared with those available in the literature and database [30,31]. The *n*-alkane series from C8 to C20 was analyzed by SPME-GC–MS. The quantification of volatile compounds was carried out using IS and calibration curves, built in the range 0.05–25 mg/kg for each volatile compound of interest. These molecules represent the major chemical classes typically present in VOOs [32]. The concentrations calculated for a total of 41 VOCs were used for the data elaboration (final data matrix 32 samples X 41 values of concentrations).

### 2.7. FGC Analysis of the Volatile Fraction

The FGC-E-nose Heracles II system (AlphaMos, Toulouse, France) employs ultra-fast gas chromatography to analyze the sample headspace. Two columns operate in parallel: a non-polar MXT5 column (5% diphenyl, 95% methylpolysiloxane, 10 m × 180 µm) and a polar MXT-1701 column (14% cyanopropylphenyl/86% dimethyl polysiloxane, 10 m × 180 µm). Each column is coupled to an FID detector, and the signal is digitized at a rate of 100 Hz. The analytical conditions applied were the same reported by Melucci et al., 2016 with some modifications related to sample conditioning temperature, namely 20 min at 40 °C and then shaking at 500 rpm, performed using an autosampler (HS 100, CTC Analytics) [33]. The 27 main peaks detected in the chromatograms obtained from the first column (MXT5) were selected through an untargeted approach (the identification of the peak was not possible), automatically integrated by the Alphasoft V12.44 software with an operator’s check to assess its suitability, and the area used for the data elaboration (final data matrix 32 samples X 27 area peaks).

### 2.8. Data Analysis

Firstly, the intensity values of volatile compounds obtained by HS-GC–IMS were used to externally validate the PLS-DA models (EV vs. noEV; L vs. noL; L vs. V; EV vs. V) developed by Valli et al., 2020 and estimate the commercial category of samples [27].

Furthermore, starting from the data matrices obtained by HS-GC–IMS (32 × 24), FGC (32 × 27), and SPME-GC–MS (32 × 41), principal component analysis (PCA) was used as an exploratory technique to visualize samples according to storage time and packaging type. In particular, four PCAs were developed, three considering separately the data sets sourced from the different instruments, and one after data fusion. In the latter case, the loading plot had to be interpreted in the context of the correlations between the original variables (the high correlation between two variables leads to two vectors that are very close to each other).

One-way analysis of variance (ANOVA) with the Tukey-HSD post hoc test was applied to sensory data with XLSTAT 2023.1.1 software (Addinsoft, New York, NY, USA) to evaluate significant differences between means of fruity positive attribute during storage.

## 3. Results

### 3.1. Sensory Analysis Results

**Figure 2:**
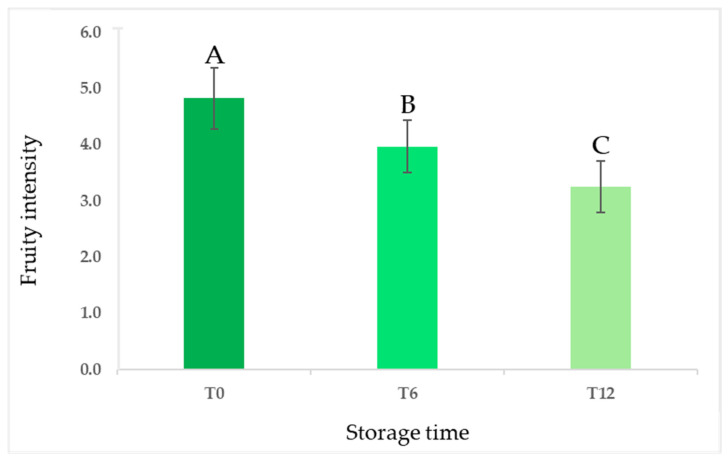
Evolution of fruity sensory intensity (expressed as means ± standard deviations) in all olive oil samples during 12 months of storage. Different letters above the bars indicate statistically significant differences as determined by one-way ANOVA (Tukey’s HSD, *p* ≤ 0.05).

The sensory analysis of the entire set of samples was conducted using an official panel test method over a one-year period of storage, with evaluations at zero, six, and twelve months [3,4]. The results indicated a decrease in the mean values of the fruity attribute, a crucial positive sensory descriptor for olive oil quality, as the storage time increased [34,35]. This decline was statistically significant and occurred progressively over 12 months, as illustrated in Figure 2.

**Figure 3:**
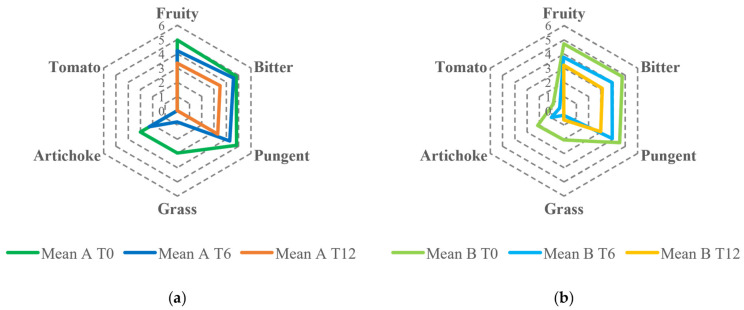
Sensory profile comparison of EVOOs obtained from olives cultivated using two agronomic systems: (**a**) integrated pest management and (**b**) organic farming, evaluated at 0, 6, and 12 months of storage.

Despite the reduction in the fruity attribute, all samples were classified as EVOO except for one, for which a rancid defect with a median score of 1.5 was perceived after 12 months of storage. In addition, the type of packaging (dark glass vs. glass bottles treated with white shielding) did not influence the evolution of the fruity intensity in olive oils during the storage period. The descriptive analysis showed the decrease over time of sensory notes resembling fresh olives, grass, and artichoke as well as bitter and pungent, especially related to secoiridoids. As shown in the spider webs in Figure 3, the intensities of the attributes were generally lower for samples produced by organic farming than those produced under the integrated pest management system. The different pungent and bitter perceptions between the samples from the two agronomic systems could be partially attributed to the varying phenolic content. Similar observations have been documented by Asami et al., 2003 and Chinnici et al., 2004 in their studies on Golden Delicious apples grown with both organic and integrated agricultural approaches [36,37].

### 3.2. HS-GC–IMS Results

The VOCs profile of samples was also analyzed during storage using the HS-GC–IMS analytical technique. From the analysis of 15 pre-selected VOCs, regions of spectral intensity of interest, comprising monomers and dimers, were identified in the heatmaps [27]. On the basis of PLS-DA calibration models built with a large database previously collected, the HS-GC–IMS data matrix was used to estimate the commercial category of samples. Specifically, combining the probability results of each model, the commercial category of samples was established (last column of Table 1): 25 samples were classified as EVOO and 1 as VOO. Six samples were identified as inconsistent (IN) (between EVOO and VOO), characterized by the inability of the models to assign a unique commercial category based on the simultaneous evaluation of the probabilities generated.

**Figure 4:**
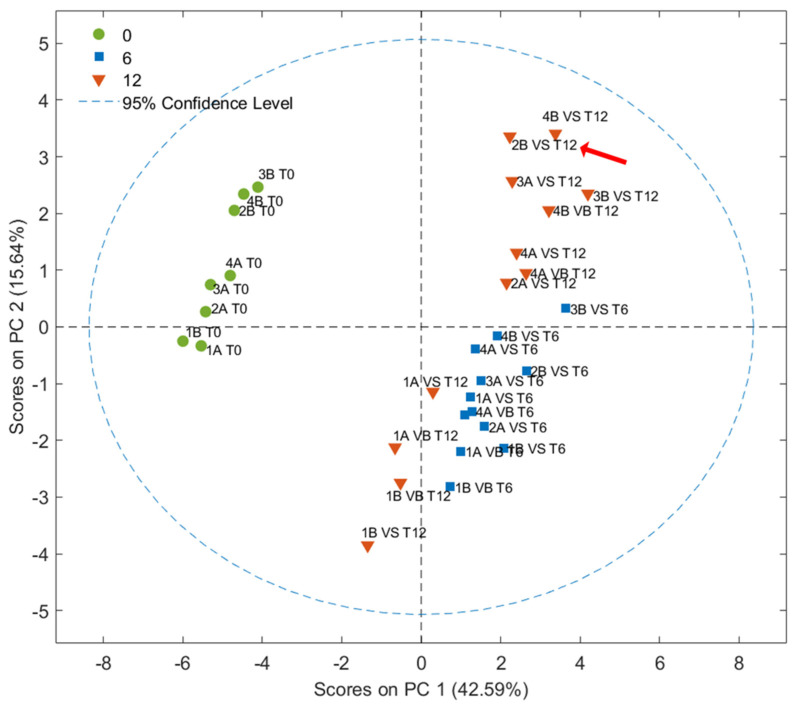
Score plots obtained from the PCA of VOOs analyzed by HS-GC–IMS at time 0 (T0) and at 6 (T6) and 12 (T12) months of storage. The sample sensory classified as VOO after one year of storage is highlighted by a red arrow.

All samples at T0 and T6 were instrumentally predicted as EVOO in agreement with the sensory classification. Considering the oils stored for 1 year, only one sample (2B_T12_VS, in bold in Table 1) was estimated as VOO, as per the sensory classification (due to the presence of a rancid defect). Six of twelve samples were estimated as IN between EVOO and VOO categories due to discrepant results in terms of the probability obtained by the four models. For example, sample 4B_T12_VS, classified as IN, showed a 79% probability of belonging to the EVOO category for the EVOO vs. no-EVOO model. However, this result was not confirmed by the PLS-DA EVOO vs. VOO model, which indicated a 33% probability of belonging to the EVOO category. PCA was employed to visualize samples according to the storage period. As shown in Figure 4, the contribution of the first and second PCs were 42.59% and 15.64%, respectively. The score plot clearly shows a separation between fresh samples (T0 in green circles) and stored samples, along the PC1. Samples stored for 6 and 12 months were separated along the PC2, except for samples collected during the first week of harvest and stored for 12 months, which showed a better state of preservation that was more similar to samples stored for only 6 months. This highlights the importance of the time of olive harvesting for the shelf life of olive oils [38]. According to Carrapiso et al., 2019, the analysis of the volatile fraction shows that the time of olive harvest influences the quality of the oil more than the type of agricultural system adopted (organic vs. conventional) [39]. Indeed, no additional clustering was observed considering the different farming systems or packaging.

### 3.3. SPME-GC–MS Results

**Figure 5:**
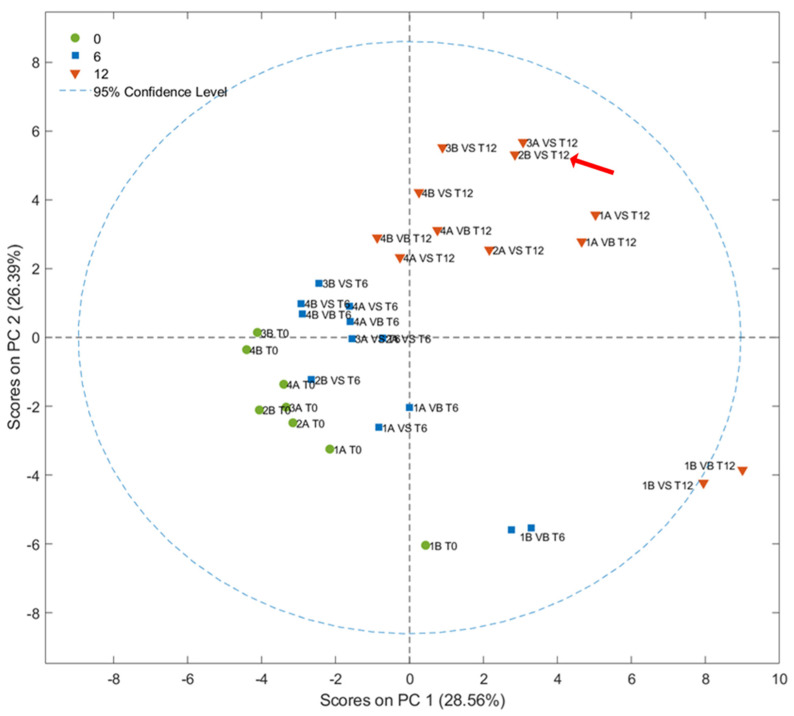
Score plot resulting from PCA of the concentrations of 41 VOCs analyzed using SPME-GC–MS in olive oils stored for 0 (T0), 6 (T6), and 12 months (T12). The sample classified as VOO after one year of storage is highlighted by a red arrow.

A total of 41 VOCs belonging to several chemical classes from different origins (LOX pathway, degradation of amino acids, sugar fermentation, lipid oxidation, etc.) were tentatively identified and quantified according to Aparicio-Ruiz et al., 2023 (Table S1) [40]. The score plot of the first two PCs (28.56%, and 26.39%) obtained by the PCA is shown in Figure 5. The fresh olive oils, represented in green, are placed in the area of the plot characterized by negative values of the two main principal components. Samples stored for 1 year, in orange, clustered on the opposite side in the area characterized by positive values of principal components. The samples stored for 6 months were distributed between these two clusters. Furthermore, the PCA results indicated that there are no relevant variations caused by the different types of packaging [41]. Similarly, when considering the time of harvest and the agronomic system employed, no clustering patterns were observed as a function of storage time.

**Figure 6:**
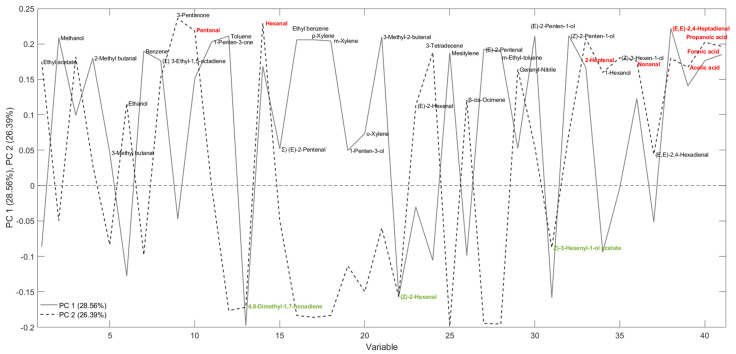
Loading plot obtained from a PCA developed using SPME-GC–MS data. Variables highlighted in red are important to the clustering of stored samples, while those in green are relevant to fresh samples.

From the loading plot analysis (Figure 6), it was possible to evaluate which variables contribute most to the sample separation. In particular, three volatile molecules colored in green and located at the bottom of the graph (4,8-dimethyl-1,7-nonadiene, (*Z*)-3-hexenal, and (*Z*)-3-hexenyl acetate) were the most important for explaining the clustering of fresh oil, considering “freshness” as the quality condition of the oil at “time zero”, i.e., immediately after milling and filtration. On the other hand, the 7 molecules in red (at the top of the graph, pentanal, hexanal, (*E*)-2-heptenal, nonanal, (*E*,*E*)-2,4-heptadienal, formic acid, and propanoic acid) were common in samples stored for 1 year and placed in the 1st quadrant of the graph and thus are reasonably markers of oxidation. For PC1, the greater contribution is due to the three markers of freshness. Specifically, (*Z*)-3-hexenal and (*Z*)-3-hexenyl acetate belong to the broader group called green leaf volatiles, consisting of molecules formed by the enzymatic oxidation of linoleic and α-linolenic acid through the action of the LOX pathway to produce aldehydes, alcohols, and esters that are responsible for the positive sensory characteristics of VOOs [42,43]. In particular, (*Z*)-3-hexen-1-ol, along with (*Z*)-3-hexenyl acetate, are the main analytes formed by (*Z*)-3-hexenal by alcohol dehydrogenase and alcohol acetyltransferase [44]. From the loading plot shown in Figure 6, it can be seen that (*Z*)-3-hexenal and (*Z*)-3-hexenyl acetate contribute to the discrimination of fresh samples, confirming what has been observed in a previous study on Brazilian EVOOs during an 8-month storage period [45].

**Figure 7:**
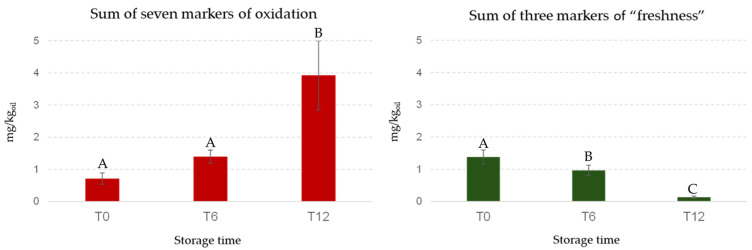
Bar graphs representing the sum of “freshness” and markers of oxidation during storage. Selected markers of lipid oxidation (red bars): pentanal, hexanal, (*E*)-2-heptenal, nonanal, (*E*,*E*)-2,4-heptadienal, formic acid, and propanoic acid. Markers of “freshness” (green bars): 4,8-dimethyl-1,7-nonadiene, (*Z*)-3-hexenal, and (*Z*)-3-hexenyl acetate. Different letters above the bars indicate statistically significant differences as determined by one-way ANOVA (Tukey’s HSD, *p* ≤ 0.05).

In contrast, PC2 was strongly influenced by pentanal, hexanal, (*E*)-2-heptenal, nonanal, (*E*,*E*)-2,4-heptadienal, formic acid, and propanoic acid. Figure 7 shows the decreasing trends of the sum of the 3 markers of “freshness” and the increase in the sum of the 7 markers of lipid oxidation during storage, selected in agreement with the literature [46]. This visual representation underscores the temporal relationship between the markers of “freshness” and those indicative of lipid oxidation [47]. As is known, the autoxidation of unsaturated fatty acids causes the formation of hydroperoxides as primary products that decompose to several secondary products as mainly saturated and unsaturated aldehydes (e.g., pentanal, hexanal, (*E*)-2-heptenal, nonanal, and (*E*,*E*)-2,4-heptadienal); the aldehydes may be then oxidized to organic acids (e.g., formic and propanoic acid) [48]. Hexanal and (*E*)-2-hexenal are very important molecules for the positive sensory attributes of fresh olive oils produced by the LOX pathway, but during storage these compounds, hexanal in particular, can also be formed by the cleavage of the same precursors (13-hydroperoxylinoleic acid 13-hydroperoxylinolenic) through autoxidation [49].

Table 2 shows the concentrations, during storage, of each marker reported in Figure 7. The aim was to investigate which markers can predict when an EVOO sample is at risk of being sensorily downgraded to VOO for rancid defect during storage. The richness of the “freshness” markers is clear, especially at time zero (T0), in samples that maintain their classification as EVOO throughout the 12-month storage period. Considering all samples and ANOVA results, a clear time trend was observed: *(Z*)-3-hexenyl acetate was detected exclusively in freshly produced samples (T0), while (*Z*)-3-hexenal showed a significant decrease over the storage period (Table 2). Additionally, 4,8-dimethyl-1,7-nonadiene was not detected in samples stored for 12 months (T12). Considering the seven markers associated with lipid oxidation, it is important to note the absence of pentanal, nonanal and (*E*)-2,4-heptadienal in T0 oils, and the significant increase in hexanal during storage. Furthermore, (*E*)-2-heptenal, propanoic acid, and formic acid were only present in samples that had been stored for one year. The only sample classified as VOO showed a characteristic evolution of volatile markers over time. This sample was distinguished by the disappearance of two fruity markers, (*Z*)-3-hexenyl acetate and 4,8-dimethyl-1,7-nonadiene (as for the others samples stored for one year), and by the highest content of (*E*)-2-heptenal that reached a concentration (0.103 mg/kg) corresponding to 20 times its odor threshold (0.005 mg/kg). This compound is recognized as one of the main contributors to the rancid defect [28]. The downgrading can be attributed to a combination of factors, including the lower fruity intensity of this sample. The synergistic effect between the reduced fruity markers and the occurrence of rancid defect contributed to its classification as a VOO.

### 3.4. FGC Results

**Figure 8:**
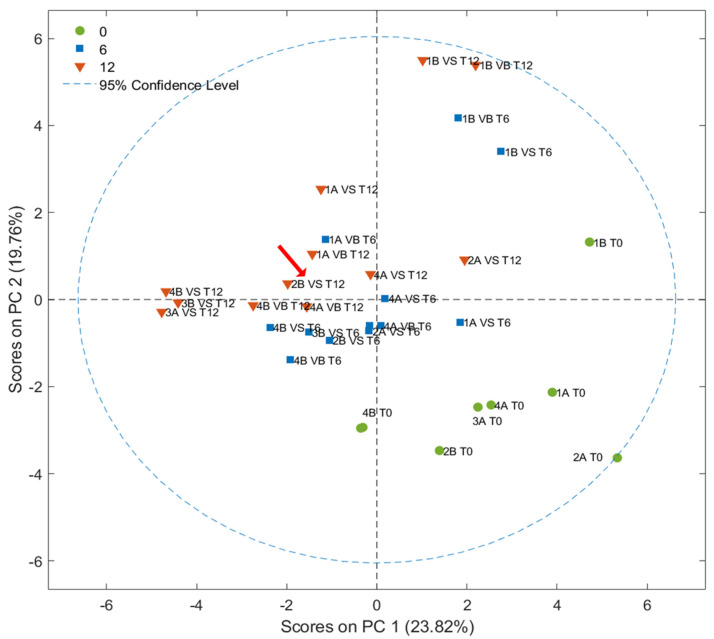
Score plot resulting from PCA developed from FGC data. The sample classified as VOO after one year of storage is highlighted by a red arrow.

**Figure 9:**
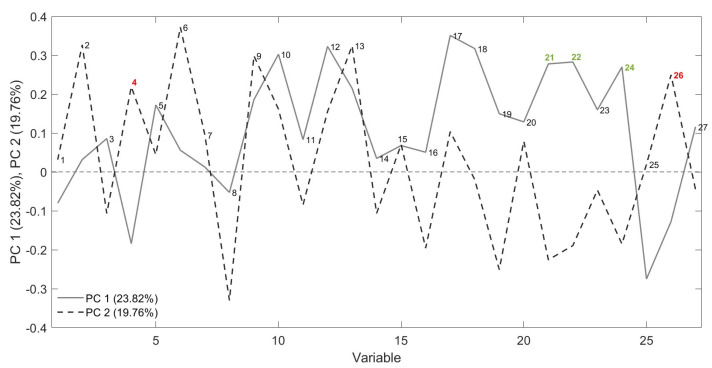
Loading plot obtained from the PCA using FGC data. Two variables highlighted in red are important to the clustering of stored samples, while the three in green are relevant to fresh samples.

Score plots (PC1 vs. PC2) obtained by PCA, developed considering 27 peak areas in the FGC first column chromatograms, are shown in Figure 8. Some discrimination according to the storage time was observed along the PCs, although it was less pronounced compared with prior results; in particular, fresh samples were mainly grouped in the fourth quadrant of the graph. In contrast, samples stored for one year were mainly placed on the opposite side in the second quadrant. The different distribution of the samples could be generated by a model that discriminates less on the basis of the previously discussed peaks (related to the fruity and rancid attributes) compared with the SPME-GC–MS technique, which considers other variables. The loadings, illustrated in Figure 9, present the most important peaks that drive the discrimination, with red indicating components that are important for clustering stored samples, while green represents those relevant to fresh samples. No distinct groupings were observed related to the type of packaging used, the agricultural system adopted, or the olive maturity index [41]. This lack of clustering suggests that, among the variables analyzed, storage time emerges as the primary discriminating factor [50].

### 3.5. Correlations Between HS-GC–IMS, FGC, and SPME/GC–MS Data

**Figure 10:**
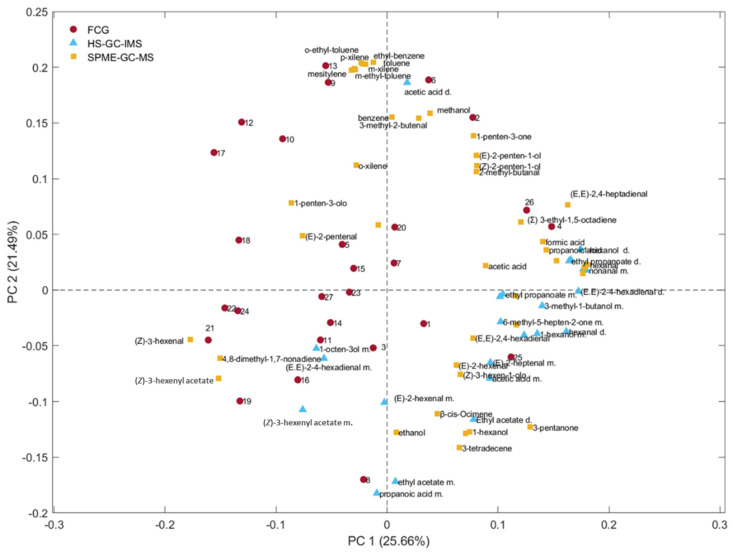
PCA of the 3 data sets (SPME-GC–MS, HS-GC–IMS, and FGC).

To understand the relationships between the models, a PCA was developed considering simultaneously the 3 data sets previously discussed. The entire matrix (autoscaled data) was composed of 92 variables (41 + 24 + 27). The loading plot shown in Figure 10 highlights the possible correlations between variables (VOCs obtained by using the three different analytical techniques). Furthermore, following the approach proposed by Melucci et al., 2016, a correlation matrix was seen between SPME-GC–MS and FGC [33]. Correlation coefficients greater than 0.7 were deemed acceptable, considering the elution order of VOCs in both analytical techniques (FGC and SPME-GC–MS; Table 3). The main aim of this elaboration was not to analyze FGC signals in depth, as the classification was untargeted, nor to consider using a data fusion approach in quality control. Instead, it was to investigate the possible differences or similarities in the variables that were most relevant to classify the oils during storage. The results of the correlation matrix are detailed in Table 3. Notably, peak number 21 exhibits a correlation with (*Z*)-3-hexenyl acetate, and a VOC of relevance to the discrimination of fresh samples by SPME-GC–MS, as illustrated in Figure 6. The correlation between peak 4 and (Σ)-3-ethyl-1,5-octadiene, identified by SPME-GC–MS, highlighted the importance of this volatile compound, generally known as a C_10_ hydrocarbon derived from 1,3-penten radicals by a secondary LOX pathway. Although it does not appear to be a primary factor in the sample clustering based on SPME-GC–MS, as demonstrated in Figure 7, the comparable distribution of this compound in the loading plots obtained with FGC and SPME-GC–MS suggests that it plays a role in discriminating samples according to storage time. Accordingly, their oxidative degradation, rather than “freshness”, would be related to the early steps of the LOX pathway. With this view, the increase in this compound, (Σ)-3-ethyl-1,5-octadiene, observed over time could plausibly be the result of the autoxidation of linolenic acid in line with the findings reported by Raffo et al., who suggested that this compound is a potential marker of the degradation of quality during storage at room temperature [51].

To provide a comprehensive view, Figure 10 presents an overall loading plot considering all samples, analytical methods, and variables. This visual representation is intended to illustrate possible relationships between the variables analyzed by targeted and untargeted approaches. The proximity of variables obtained using different techniques suggests a positive correlation between them. As an example, (*Z*)-3-hexenyl acetate, a compound identified by SPME-GC–MS and HS-GC–IMS, is located close to peak 21, a variable detected by FGC. Similarly, methanol, identified by SPME-GC–MS, is positioned close to peak 2. However, it was not possible to confirm this using HS-GC–IMS because methanol was not included among the 15 volatile markers analyzed for VOO classification in this study. The correlation matrix reported in Table 3 highlights the relationships among the data obtained by SPME-GC–MS and FGC; in particular, (*Z*)-3-hexenyl acetate in SPME-GC–MS correlates with an FGC retention time, an important marker for clustering fresh oils.

## 4. Conclusions

This study investigated the changes in monovarietal VOOs during storage (0, 6, and 12 months) by combining sensory analysis with targeted (SPME-GC–MS), screening targeted (HS-GC–IMS), and untargeted (FGC) approaches to the analysis of VOCs. Sensory analysis results showed a decrease in the fruity attribute during storage, in alignment with the decline in volatile compounds related to this positive attribute (4,8-dimethyl-1,7-nonadiene, (*Z*)-3-hexenal, and (*Z*)-3-hexenyl acetate), and linked with the “freshness” of EVOO, that is, as already specified to its quality at time zero, i.e., immediately after the milling and filtration. Almost all samples were classified as EVOO by a panel test throughout the entire storage period. However, one sample exhibited a rancid defect after 12 months and was thus classified as VOO, in accordance with the estimation attributed by the PLS-DA models with the HS-GC–IMS screening method. Furthermore, with the same screening method and predictive models, 6 samples stored for 12 months, despite being classified as EVOO by a panel test, were classified as “inconsistent” (between EVOO and VOO), which can be interpreted as a “red flag”, i.e., a risk of declassification after 12 months. To gain additional information, targeted SPME-GC–MS analysis was used to identify, quantify, and select the VOCs whose concentrations changed more during the storage period, being reasonably considered as the main variables of “freshness” and lipid oxidation. A PCA of SPME-GC–MS data, clustering samples based on the storage time, allowed for the selection of markers in fresh oils (4,8-dimethyl-1,7-nonadiene, (*Z*)-3-hexenal, and (*Z*)-3-hexenyl acetate) and in oils stored for 12 months (pentanal, hexanal, (*E*)-2-heptenal, nonanal, (*E*,*E*)-2,4-heptadienal, formic acid, and propanoic acid). In this study, the variable most responsible for the downgrade of the only sample from EVOO to VOO after 12 months of storage was associated with a volatile marker well known to be linked to lipid oxidation. In fact, the concentration of (*E*)-2-heptenal in this sample was approximately twenty times higher than its odor threshold. Untargeted FGC analysis, characterized by its rapid execution time, by PCA, clustered samples according to storage time, but with a lower effectiveness than the other techniques employed herein. However, the PCA results obtained from the three analytical techniques show that the samples are mainly grouped according to storage time, rather than by other factors evaluated in the study, such as agronomical practices, maturity index, and packaging type.

Overall, an integrated approach, combining sensory evaluation with targeted and untargeted analysis of the volatile fraction, can help in identifying the molecules (in a targeted approach), regions of a chromatogram, or regions of a heatmap (in an untargeted one) to be considered when predicting the changes in quality occurring during the storage of olive oil, thus contributing to the indication of the correct “best before date” on the label.

## Figures and Tables

**Table 1:** Results, in terms of probability values, of HS-GC–IMS analysis of the entire sample set applying the four previously built PLS-DA models and resulting olive oil commercial categories.

Sample	EVOO vs. No-EVOO	LOO vs. No-LOO	LOO vs. VOO	EVOO vs. VOO	Resulting Category
1A_T0_VS	0.79	0.13	0.07	0.86	EVOO
1B_T0_VS	0.77	0.11	0.06	0.89	EVOO
2A_T0_VS	0.73	0.31	0.17	0.71	EVOO
2B_T0_VS	0.85	0.31	0.11	0.89	EVOO
3A_T0_VS	0.73	0.31	0.18	0.70	EVOO
3B_T0_VS	0.88	0.18	0.06	0.88	EVOO
4A_T0_VS	0.74	0.31	0.18	0.69	EVOO
4B_T0_VS	0.88	0.31	0.18	0.85	EVOO
1A_T6_VS	0.82	0.08	0.07	0.93	EVOO
1B_T6_VS	0.87	0.07	0.08	0.96	EVOO
1A_T6_VB	0.80	0.10	0.06	0.88	EVOO
1B_T6_VB	0.89	0.07	0.11	0.94	EVOO
2A_T6_VS	0.76	0.16	0.07	0.81	EVOO
2B_T6_VS	0.86	0.13	0.06	0.92	EVOO
3A_T6_VS	0.78	0.31	0.18	0.78	EVOO
3B_T6_VS	0.91	0.08	0.08	0.92	EVOO
4A_T6_VS	0.78	0.31	0.18	0.74	EVOO
4B_T6_VS	0.90	0.28	0.08	0.90	EVOO
4A_T6_VB	0.80	0.25	0.11	0.79	EVOO
4B_T6_VB	0.90	0.24	0.08	0.92	EVOO
1A_T12_VS	0.64	0.21	0.51	0.35	IN
1B_T12_VS	0.85	0.09	0.23	0.92	EVOO
1A_T12_VB	0.67	0.14	0.23	0.28	IN
1B_T12_VB	0.84	0.08	0.18	0.88	EVOO
2A_T12_VS	0.64	0.10	0.06	0.47	IN
**2B_T12_VS**	0.43	0.16	0.40	0.21	VOO *
3A_T12_VS	0.55	0.07	0.07	0.19	IN
3B_T12_VS	0.88	0.07	0.18	0.71	EVOO
4A_T12_VS	0.74	0.19	0.10	0.62	EVOO
4B_T12_VS	0.79	0.07	0.10	0.33	IN
4A_T12_VB	0.59	0.08	0.06	0.29	IN
4B_T12_VB	0.80	0.12	0.07	0.51	EVOO

* This sample was the only sample classified as VOO also by panel test (rancid defect with a median score of 1.5).

**Table 2:** Concentrations (mean values of samples analyzed at T0, T6, T12) of the seven oxidative markers and three “freshness” markers monitored during storage. The letters indicate significant differences (one-way ANOVA, Tukey’s HSD, *p* < 0.05) among the samples.

Volatile Markers	T0	T6	T12
	Concentration (mg/kg)		
pentanal	0.00 ^a^	0.10 ^b^	0.22 ^c^
hexanal	0.71 ^a^	1.18 ^b^	2.20 ^c^
(*E*)-2-heptenal	0.00 ^a^	0.00 ^a^	0.056 ^b^
nonanal	0.00 ^a^	0.05 ^b^	0.06 ^b^
(*E*,*E*)-2,4-heptadienal	0.00 ^a^	0.07 ^b^	0.24 ^c^
formic acid	0.00 ^a^	0.00 ^a^	1.09 ^b^
propanoic acid	0.00 ^a^	0.00 ^a^	0.07 ^b^
4,8-dimethyl-1,7-nonadiene	0.74 ^a^	0.69 ^a^	0.00 ^b^
(*Z*)-3-hexenal	0.60 ^a^	0.28 ^b^	0.13 ^c^
(*Z*)-3-hexenyl acetate	0.04 ^a^	0.00 ^b^	0.00 ^b^

**Table 3:** Correlation matrix results (r > 0.7) between SPME-GC–MS and FGC data. FGC retention time is expressed in seconds.

VOC	Peak Number	FGC Retention Time	r
methanol	2	20.57	0.77
ethanol	3	22.63	0.72
(Σ) 3-ethyl-1,5-octadiene	4	24.46	0.72
(*Z*)-3-hexenyl acetate	21	95.76	0.76

## Data Availability

The original data used in this study are openly available in AMSActa at the following DOI: https://doi.org/10.6092/unibo/amsacta/8136 (accessed on 20 May 2025).

## Supplementary Materials

The following supporting information can be downloaded at: https://www.mdpi.com/article/10.3390/foods14111884/s1, Table S1: tentative identification of the 41 volatile compounds by SPME-GC–MS in olive oils stored at 0 (T0), 6 (T6), and 12 months (T12).

## Abbreviations

The following abbreviations are used in this manuscript:

HS-GC–IMS	Headspace gas chromatography–ion mobility spectrometry
FGC	Flash gas chromatography
EU	European Union
EVOO	Extra virgin olive oil
VOO	Virgin olive oil
IN	Inconsistent
LOO	Lampante olive oil
IOC	International Olive Council
LRI	Linear retention indices
SPME-GC–MS	Solid-phase microextraction-gas chromatography–mass spectrometry
FID	Flame ionization detector
PCA	Principal Component Analysis
PLS-DA	Partial least squares discriminant analysis
LOX	Lipoxygenases
VOC	Volatile compound
PDO	Protected Designation of Origin
