# Supplementary material for: Changes in the Volatile Profile, Fruity Flavor, and Rancidity in Virgin Olive Oils During Storage by Targeted and Untargeted Analytical Approaches
Source: Foods. 2025 May 26;14(11):1884. doi: 10.3390/foods14111884 (PMC12155163; doi:10.3390/foods14111884)
Supplement: Supplementary file 1 [file foods-14-01884-s001.zip › Supplementary materials.docx]

**Supplementary Materials:**

**Table S1**: tentative identification of the 41 volatile compounds by SPME-GC-MS in olive oils stored at zero (T0), six months (T6), and twelve months (T12).

| Volatile compounds | LRI |
| --- | --- |
| ethyl acetate | 903 |
| methanol | 909 |
| 2,3-epoxy-2-methyl pentane | 912 |
| 2-methyl-butanal | 923 |
| 3-methyl-butanal | 927 |
| ethanol | 945 |
| benzene | 952 |
| (Σ) 3-ethyl-1,5-octadiene | * |
| 3-pentanone | 990 |
| pentanal | 992 |
| 1-penten-3-one | 1035 |
| toluene | 1053 |
| 4,8-dimethyl-1,7-nonadiene | 1094 |
| hexanal | 1095 |
| (E)-2-pentenal | 1122 |
| ethyl-benzene | 1141 |
| p-xilene | 1149 |
| m-xilene | 1155 |
| 1-penten-3-olo | 1173 |
| o-xilene | 1199 |
| 3-methyl-2-butenal | 1215 |
| (*Z*)-3-hexenal | 1218 |
| (*E*)-2-hexenal | 1235 |
| 3-tetradecene | 1245 |
| mesitylene | 1261 |
| β-cis-ocimene | 1266 |
| o-ethyl-toluene | 1279 |
| m-ethyl-toluene | 1299 |
| geranyl-nitrile | 1321 |
| (*E*)-2-penten-1-ol | 1327 |
| (*Z*)-3-hexenyl acetate | 1333 |
| (*Z*)-2-penten-1-ol | 1335 |
| 2-heptenal | 1344 |
| 1-hexanol | 1366 |
| (*Z*)-3-hexen-1-olo | 1399 |
| nonanal | 1426 |
| (*E,E*)-2,4-hexadienal | 1449 |
| (*E,E*)-2,4-heptadienal | 1580 |
| acetic acid | 1587 |
| propanoic acid | 1675 |
| formic acid | 1785 |

*LRI of (Σ) 3-ethyl-1,5-octadiene: 965, 973, 1017and 1033
